# DeepEdit: single-molecule detection and phasing of A-to-I RNA editing events using nanopore direct RNA sequencing

**DOI:** 10.1186/s13059-023-02921-0

**Published:** 2023-04-17

**Authors:** Longxian Chen, Liang Ou, Xinyun Jing, Yimeng Kong, Bingran Xie, Niubing Zhang, Han Shi, Hang Qin, Xuan Li, Pei Hao

**Affiliations:** 1 Key Laboratory of Synthetic Biology, CAS Center for Excellence in Molecular Plant Sciences, Institute of Plant Physiology and Ecology, Chinese Academy of Sciences, Shanghai, China; 2 Key Laboratory of Molecular Virology and Immunology, Institut Pasteur of Shanghai, Chinese Academy of Sciences, Shanghai, China; 3 Department of Genetics and Genomic Sciences and Icahn Institute for Genomics and Multiscale Biology, Icahn School of Medicine at Mount Sinai, New York, NY USA; 4 University of Chinese Academy of Sciences, Beijing, China

**Keywords:** RNA editing, Nanopore direct RNA sequencing, Phasing, Neural network model

## Abstract

Single-molecule detection and phasing of A-to-I RNA editing events remain an unresolved problem. Long-read and PCR-free nanopore native RNA sequencing offers a great opportunity for direct RNA editing detection. Here, we develop a neural network model, DeepEdit, that not only recognizes A-to-I editing events in single reads of Oxford Nanopore direct RNA sequencing, but also resolves the phasing of RNA editing events on transcripts. We illustrate the robustness of DeepEdit by applying it to *Schizosaccharomyces pombe* and *Homo sapiens* transcriptome data. We anticipate DeepEdit to be a powerful tool for the study of RNA editing from a new perspective.

**Supplementary Information:**

The online version contains supplementary material available at 10.1186/s13059-023-02921-0.

## Background

RNA editing is a universal and critical post-transcriptional event discovered in diverse life forms [1–3]. The deamination of adenosine, which converts adenosine to inosine (A-to-I), is the most abundant RNA editing event in metazoan [4–6]. This process is catalyzed by adenosine deaminase acting on RNA (ADAR) family on double-stranded RNA substrates [7–9]. With inosines being read as guanines (Gs) in translation, A-to-I RNA editing events are prone to alter the protein sequencings and create versatility in protein products [10]. To identify RNA editing sites, previous approaches were mainly developed on short-read whole transcriptome sequencing by analyzing the single-nucleotide variants (SNVs) between mapped reads and the genome reference [5]. However, this method faces several challenges, including not being able to determine the phasing information of edited bases (whether multiple edited bases co-locating on the same transcripts), false-positive editing sites identified due to the mis-mapping of short-length reads, and computational complexity to decipher the relations of RNA editing events with other post-transcriptional events, e.g., alternative splicing [11, 12]. The advent of Oxford Nanopore Technology (ONT) provides a solution by direct RNA sequencing without reverse transcription or PCR amplification [13]. By recording the electrical signal changes, direct RNA sequencing has been applied to the identification of RNA modifications, including N6-methyladenosine (m6A) [14–17] and others [18–20]. Encouragingly, this technology was applied to RNA editing identification and achieved site-level RNA editing prediction by generating a machine-learning model with the nanopore reads [21]. Nonetheless, the current model cannot detect editing events on a single nanopore read (read-level), let alone resolving the phasing information of RNA editing events on a single transcript. In this study, to differentiate RNA editing events among different nanopore reads, we developed a neural network model, DeepEdit, that can identify A-to-I editing events on single nanopore reads and determine the phasing information on transcripts through nanopore direct RNA sequencing.

## Results and discussion

**Fig. 1:**
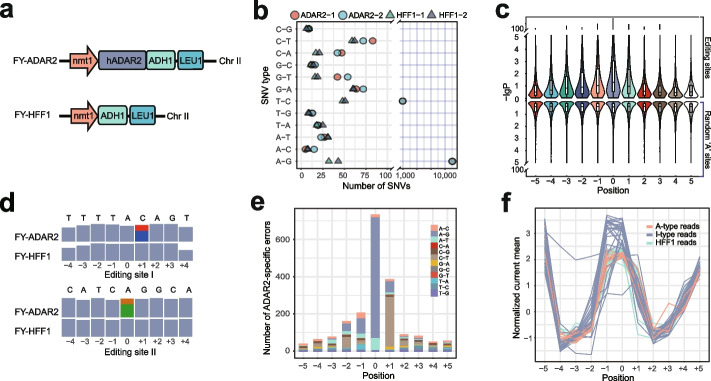
Characterization of A-to-I RNA editing events on nanopore reads. **a** Construction of *Schizosaccharomyces pombe* strains with de novo RNA editing machinery. Top, introduction of human hADAR2 gene into *S. pombe* chromosome II through homologous recombination. Bottom, the negative strain without hADAR2 coding sequence. *mnt1*, transcription promoter. hADAR2, coding sequence. ADH1, transcription terminator. LEU1, insertion locus on chromosome II. **b** Confirmation of RNA editing events in positive FY-ADAR2 and negative FY-HFF1 strains by Illumina datasets, shown with counts of single nucleotide variations (SNVs) on *x*-axis and SNV types on *y*-axis. ADAR2-1 and ADAR2-2 are two replicates of FY-ADAR2 strain. HFF1-1 and HFF1-2 are two replicates of FY-HFF1 strain. **c** The edited adenine sites had a significant upward *p*-value shift at the editing sites as well as nearby bases. Violin plots show the *p*-values (-log10 transformed) of context bases from − 5 to + 5 around the edited bases (position 0). Top, *P* values of editing domains. Bottom, *P* values of random “A” domains. **d** Examples of ADAR2-specific errors (ASEs) around editing positions. Bases from − 4 to + 4 surrounding the editing sites are shown, with the correct reference bases displayed on the top. The gray bars denote reads coverage. The colored bars denote the ratios of different bases for the error sites. Red, thymine. Blue, cytosine. Orange, guanine. Green, adenine. **e** Frequencies of different types of ADAR2-specific errors, shown with bases from − 5 to + 5 around editing sites. **f** Electrical signal separation of different read types. Red lines denote A-type reads from FY-ADAR2 samples. Blue lines denote I-type reads from FY-ADAR2 samples. Green lines denote negative control reads from FY-HFF1 samples

To achieve single-molecule (read-level) detection of A-to-I RNA editing events on in vivo transcribed RNAs, we constructed *Schizosaccharomyces pombe* strains with de novo RNA editing machinery for nanopore direct RNA sequencing. *S. pombe* lacks the ADAR-mediated A-to-I RNA editing system, which provides us with a “clean” background to study changes of nanopore electrical signals caused by A-to-I RNA editing. We introduced the human adenosine deaminase acting on RNA type 2 (hADAR2) gene into *S. pombe* chromosome II, resulting in a strain capable of A-to-I RNA editing, namely FY-ADAR2 (Fig. 1a and Additional file 1: Fig. S1a, b). We also constructed a control strain (FY-HFF1), which contains the nmt1 promoter and ADH1 terminator but has no hADAR2 coding sequence. The PCR and western blot results showed the correct introduction and successful expression of hADAR2 protein in FY-ADAR2 (Additional file 1: Fig. S1c, d).

To determine whether the hADAR2 protein can edit RNA in yeast cells, total RNA was extracted from FY-ADAR2 and FY-HFF1 and sequenced with Illumina Novaseq. The Illumina reads were mapped to the reference transcriptome for analysis. For FY-ADAR2, 11,615 and 11,496 A-to-G change sites, and 967 and 930 T-to-C change sites were detected in replicate experiments (Fig. 1b). Note, these T-to-C nucleotide changes were due to A-to-I RNA editing events happening on the transcripts reverse complementary to reference mRNAs. This data demonstrated the validity of hADAR2-mediated A-to-I RNA editing in the yeast strain FY-ADAR2. In contrast, for FY-HFF1 samples, only tens of A-to-G change sites were found, representing a background noise of false-positive sites. We further defined a high-confident subset of A-to-I RNA editing, including 6965 sites, from FY-ADAR2 by requiring an editing ratio greater than 0.1 (see the “Methods” section). To validate these editing sites, 10 sites were randomly selected for RT-PCR and Sanger sequencing analysis, and all of them were confirmed (Additional file 1: Fig. S2).

To check the effect of A-to-I conversion on nanopore electrical signals, we performed direct RNA sequencing on mRNA from FY-ADAR2 and FY-HFF1 on a GridION platform with R9.4 flowcells. A total of 2,328,631 reads from FY-HFF1 and 4,224,232 reads from FY-ADAR2 were obtained. To investigate the electrical signal shift on A-to-I RNA editing sites, the normalized electrical signals of editing sites in FY-ADAR2 and those of the corresponding sites in FY-HFF1 were compared using the Kolmogorov–Smirnov test (KS-test). As a comparison, the electrical signals of random “A” sites in FY-ADAR2 samples were tested against those of the corresponding sites in the FY-HFF1 sample, which was regarded as the background electrical noise between the two samples. While the *P* value distribution for the comparison of the random “A” sites (random “A” sites in FY-ADAR2 vs “A” counterparts in FY-HFF1) is flat, the edited “A” sites (edited “A” sites in FY-ADAR2 vs “A” counterparts in FY-HFF1) have a significant difference at the edited sites and nearby bases (− 3, − 2, − 1, and + 1) (Fig. 1c). The results revealed that A-to-I RNA editing events can cause an electrical signal shift in nanopore native RNA sequencing. Besides, they also induced current signal changes at sites adjacent to the editing bases. Notably, the signal from the edited bases is most affected, whereas the electrical signals of − 1, − 2, − 3, and + 1 sites are affected to a smaller degree. Other sites further away saw little or no effect.

To make it possible to detect the editing events on adenosine residues in single nanopore sequencing reads, we first separated the edited and unedited reads for each editing site before model training. We found the base-calling errors were associated with A-to-I editing. The signal shifts caused by A-to-I editings may hinder the resolution by base-callers due to the unexperienced electrical signal values [14]. We observed frequent base-calling errors on editing positions in FY-ADAR2 samples and very few in FY-HFF1 counterparts (Fig. 1d), suggesting that these ADAR2-specific errors (ASEs) were likely related to the presence of editing events on A-to-I edited reads. We further plotted ASE density for the A-to-I editing sites as well as randomly selected “A” sites. ASEs were frequently found at edited sites and nearby bases, which mostly concentrate at − 1 to + 1 sites (Fig. 1e and Additional file 2: Table S1). In comparison, only baseline-level and randomly distributed errors were observed around random “A” sites (Additional file 1: Fig. S3a and Additional file 2: Table S2), indicating a correlation between the ASEs and the RNA editing events. Therefore, ASEs could be used as a unique feature to distinguish inosine-containing RNA molecules from unedited ones.

Based on these findings, we separated the nanopore reads mapped on editing sites in FY-ADAR2 samples into two groups, A-to-I edited reads (I-type reads) and unedited reads (A-type reads), for subsequent model training (Additional file 1: Fig. S3b and the “Methods” section). To evaluate the effectiveness of the read-separation, we plotted the electrical signals of I-type and A-type reads as well as those from FY-HFF1 negative control (HFF1 reads) (Fig. 1f). The results showed that the electrical signals of I-type reads are significantly different at the editing sites or nearby positions compared to that of HFF1 reads but converged at positions further away. In comparison, the signals of A-type reads on the editing sites are similar to that of HFF1 reads on the same position. In summary, the A-to-I conversion shifts the electrical signals on editing sites. These shifted signals may lead to non-random base-calling errors. These specific errors, ASEs, on and around editing sites could be used for read separation. The annotated I-type reads and HFF1 reads were used as positive and negative controls, respectively, for the subsequent model training (see the “Methods” section).

**Fig. 2:**
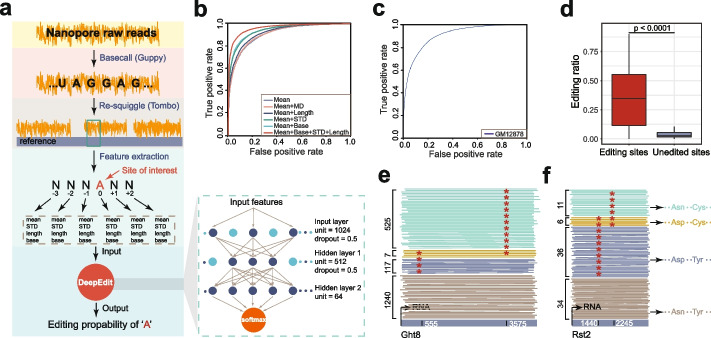
Prediction of A-to-I RNA editing events on single nanopore reads by DeepEdit. **a** DeepEdit workflow. The DeepEdit pipeline involves 3 main steps: (1) data processing, which includes base-calling and re-squiggle; (2) feature extraction, where features of bases from − 3 to + 2 around editing sites were extracted; (3) neural network modeling, in which the extracted features are fed into a neural network model for prediction. Please refer to the “Methods” section for a detailed description. **b** Performance of different feature combinations in *S. pombe*, shown with receiver operator characteristic (ROC) curves. The ROC curves demonstrate the prediction performance using different combinations of normalized electrical signal means (Mean), mean deviations between adjacent bases (MD), standard deviations (STD), the number of raw signal values (Length), and base type (Base) of editing regions. **c** ROC curve for the read-level prediction of A-to-I RNA editing events in human. **d** Editing ratios predicted by DeepEdit for known edited and unedited sites in human datasets. **e** Phasing of long-spanned RNA editing sites in *S. pombe* using DeepEdit. Examples of RNA molecules (reads) are shown. Red asterisks denote the editing events identified by DeepEdit. Numbers of different read types are shown on the left. Colored lines denote RNA molecules with distinct editing status. Gray lines denote unedited RNA molecules. Dark blue and green lines denote RNA molecules edited on either site. Yellow lines denote RNA molecules edited on both sites. **f** Similar as **e**, but for the Rst2 gene. Examples of RNA molecules (reads) are shown. Potential amino acids coded on editing sites are illustrated on the right

To detect RNA editing events on nanopore RNA sequencing reads, we designed a fully connected neural network model, DeepEdit, which takes advantage of the raw electrical signal features flanking the editing sites (Fig. 2a and the “Methods” section). To train the model, a total of 40,823 I-type reads from FY-ADAR2 and randomly chosen 47,757 HFF1 reads were used as the positive and negative controls. To obtain the best performance of the neural network model, we selected and compared the performances of five individual or combined raw features, including normalized electrical signal means (Mean), mean deviations between adjacent bases (MD), standard deviations (STD), the number of raw signal values (Length), and base type (Base) (Fig. 2b and the “Methods” section). Independent cross-validation showed that combinations of raw features, except MD, greatly improved the performance. The combination of Mean, Base, STD, and Length showed the best performance (AUC score: 0.9653) (Fig. 2b and Additional file 1: Fig. S4a-f). Therefore, we applied these specific combined features of the 6-mers spanning the RNA-editing sites (− 3 to + 2) in DeepEdit and finally got a model for RNA editing events detection in nanopore RNA sequencing reads with high accuracy in *S. pombe*.

To evaluate the cross-species performance of DeepEdit trained on *S. pombe* data, we applied DeepEdit on a nanopore direct RNA sequencing data from human cell line GM12878 [22]. For comparison, RNA editing sites from matched Illumina data were used as the benchmark. To reliably validate the read-level performance of DeepEdit on the human RNA nanopore sequencing data, we chose completely edited and unedited sites for validation (see the “Methods” section). The nanopore reads mapped to the edited sites were labeled as positive reads, and those mapped to the unedited sites were labeled as negative reads. Application of DeepEdit on a total of 79,426 nanopore reads achieved an AUC score of 0.9076 (Fig. 2c and Additional file 1: Fig. S4g). Consistently, when applying DeepEdit to predict the editing status across genomic sites, the editing ratio of editing sites was significantly higher (*P* < 0.0001) than those of unedited sites (Fig. 2d), indicating a fairly good performance of DeepEdit on distinguishing editing RNA molecules from unedited ones in human. Furthermore, we collected two atlases of RNA editing sites that were reported by previously published studies and conducted comparisons with the sites detected by our approach. Our method achieved overlap rates of 56.32% (Additional file 2: Table S4) with the first study [23] and 54.98% (Additional file 2: Table S5) with the second study [24] (see the “Methods” section). Considering previous studies have shown overlap rates of 48.87% between Ramaswami et al. [23] and Li et al. [25], or 56.48% between Ramaswami et al. [23] and Peng et al. [24], our results represent a comparable overlap rate with previous studies. All analyses indicate broad applications of DeepEdit trained with *S. pombe* data on different species.

Our long-read approach offers several advantages over the short-read methods. First, it is achievable to detect editing sites in the repetitive elements and hyper-edited regions. For example, we screened the Alu repeat regions on 10 transcripts and detected 104 editing sites (Additional file 2: Table S6), of which 96 sites (92.30%) were annotated as potential editing sites in the REDIportal database. In contrast, the short-read method missed a considerable part of sites with only 50 sites detected, due to the difficulty of short-read alignment in these hyper-edited regions (Additional file 1: Fig. S5). Second, with the advantage of the long-read length and single-molecule resolution by DeepEdit, we were able to report the phasing information of A-to-I RNA editing events on RNA molecules. For instance, *S. pombe* RNA transcripts from Ght8 harbored two mutually exclusive editing events, although these two sites (residues 555 and 3575) spanned a long distance (~ 3 kb) (Fig. 2e). Among the total 1882 mapped nanopore reads, only 7 (0.37%) molecules were edited simultaneously on both residues. 117 nanopore reads were predicted to be edited solely on residue 555, and 525 nanopore reads were edited solely on residue 3575.

RNA editing plays important roles in multiple human diseases, including neurological disorders [26–28] and cancers [29, 30]. Identification and characterization of RNA editing events are crucial to understanding molecular regulations such as protein re-coding and secondary structural changes. For example, in Alzheimer’s disease, the serotonin receptor HTR2C showed a reduced level of A-to-I RNA editing [28]. There are five potential editing sites on a certain transcript, which potentially lead to 24 different protein isoforms. The short-read method may be unable to estimate the actual protein isoforms in similar cases if the editing sites span a long distance. Using long reads at single-molecule resolution, DeepEdit could identify the editing events on each transcript isoform, thus directly revealing the potential protein products (Fig. 2f). The ability to phase RNA editing events provides new opportunities for DeepEdit in RNA editing research, including characterizing possible RNA secondary structure changes induced by editing events (Additional file 1: Fig. S6).

A recent study generated a model (Dinopore) for the site-level prediction of RNA editing events with aggregate nanopore read profiles [21]. Their model returns a prediction of whether the sites were edited, but was not able to detect editing events in single nanopore sequencing reads. In contrast, DeepEdit is capable of single-molecule (read-level) detection of RNA editing events. The single-molecule resolution provides DeepEdit advantage to identify the editing events independent of editing ratios, e.g., in the lowly edited Alu repeat regions (Additional file 2: Table S6). Furthermore, DeepEdit can determine the phasing information of editing events, which is not possible with the site-level nanopore method such as Dinopore, or Illumina short-read methods.

It is worth mentioning, DeepEdit has its own limitations. To generate the training datasets, DeepEdit required de novo construction of RNA editing machinery for comparative nanopore direct RNA sequencing. While we have demonstrated a successful application of DeepEdit to human cell lines using datasets trained from *S. pombe* (Fig. 2c, d), it is still challenging to predict RNA editings in specific regions, especially those unique to the human genome. To address this issue, we plan to create additional training datasets from multiple species in the future, which will enable DeepEdit to predict RNA editing across more genome contexts.

## Conclusions

In this study, we demonstrated that A-to-I RNA editing events can alter electrical signals spanning the edited residues, allowing us to differentiate RNA editing events among different nanopore reads. We developed a neural network model, DeepEdit, which utilizes combined electrical signal features, to detect RNA editing events on single RNA sequencing reads. Our results showed that DeepEdit can robustly detect A-to-I editing events on nanopore RNA sequencing reads from different species, including *S. pombe* and *H. sapiens*, enabling us to phase editing events and gain new insights into the post-transcriptional regulation of RNA. We believe that DeepEdit will be a valuable tool for the RNA editing research community and help advance our understanding of RNA-editing-related human diseases. A detailed protocol and software for DeepEdit are available at https://github.com/weir12/DeepEdit.

## Methods

### Plasmids and yeast strains

#### Plasmids

The hADAR2 coding sequences were amplified from cDNA of 293 T cells with primer hADAR2-P5 and hADAR2-P3 (Additional file 2: Table S3). The plasmid pDUAL-HFF1-hADAR2 was constructed by inserting the hADAR2 coding sequences into the NheI/BglII site of plasmid pDUAL-HFF1 (RIKEN BioResource Center, Ibaraki, Japan). PCR was performed using KOD FX DNA polymerase (TOYOBO). DNA fragments were digested using restriction endonucleases and ligated using T4 DNA ligase (New England Biolabs). *Escherichia coli* DH5α was used for molecular cloning. Plasmids were extracted from hosts using the Plasmid Mini Kit I (OMEGA).

#### Transformation of yeast strains

*S. pombe* strain FY7652 (h-leu1-32 ura4-D18) (National BioResource Project, Osaka, Japan) was used to generate derived strains by transformation with various plasmids. FY-HFF1 and FY-ADAR2 strains were generated by transformation using the Lithium Acetate/PEG/Heat shock method [31]. Plasmids (pDUAL-HFF1-hADAR2 and pDUAL-HFF1) were digested with the NotI restriction enzyme and treated with FastAP thermosensitive alkaline phosphatase (Thermo Scientific). Transformation was carried out with 500 ng of gel-recovered linear DNA (Gel Extraction Kit from OMEGA). Transformants were selected on EMM medium supplemented with 50 μg/mL uracil and verified for chromosomal integration by PCR with primer set ADHterm-F and leu1-R [32]. Sequences of all primers are listed in Additional file 2: Table S3.

#### Cultivation of yeast strains

*S. pombe* strains FY-ADAR2 and FY-HFF1 were plated in EMM + ura plate [33] and grown for 4 days. Colonies were picked and cultured in 3-mL EMM medium and grown until mid-log phase. Harvested cells were inoculated in 20 mL of EMM medium with an initial optical density (OD_600_) of 0.1. Cells in the mid-log phase were harvested for DNA and RNA extraction.

### Illumina sequencing

Total RNA from FY-HFF1 and FY-ADAR2 strains was extracted using TRIZOL reagent (Invitrogen) according to the manufacturer’s instructions. RNA integrity (RIN) was assessed using an Agilent 2100 Bioanalyzer. mRNA was purified from total RNA using poly-T oligo, and sequencing libraries were generated using TruSeq RNA Library Preparation Kit (Illumina, USA). The libraries were sequenced on an Illumina Novaseq platform and 150 bp PE reads were generated (Nextomics Biosciences, Wuhan, China).

### Analysis of Illumina sequencing data

#### *S. pombe* RNA-seq datasets

The raw Illumina sequencing data were first trimmed from 5′ end using Fastx_trimmer (version 0.0.13) with options -f11 -Q33. Replicate reads were removed using FastUniq [34], and then sickle (version 1.33 https://github.com/najoshi/sickle) was used to remove the low-quality reads with options -l 50 -n -q 30. The workflow for the identification of A-to-I RNA editing sites is as described [3]. Briefly, the filtered Illumina reads were first aligned to the reference transcriptome of *S. pombe* (GCF_000002945.1_ASM294v2_rna) downloaded from NCBI using Hisat2 [35] (version 2.0.5). Second, the base variants were called using Samtools [36] mpileup package (version 1.10, options -Q 20 -ugf) and bcftools [37] (options -vc). Third, high-confidence A-to-I RNA editing sites were identified using the criteria: (1) retaining only A-to-G SNVs occurred both in FY-ADAR2-1 and FY-ADAR2-2 samples; (2) removing the A-to-G SNVs occurred in FY-HFF1-1 or FY-HFF1-2; (3) removing the sites that overlapped with genomics SNPs; (4) coverage depth ≥ 50 and editing ratio ≥ 0.1 in all FY-ADAR2 samples.

#### *H. sapiens* RNA-seq datasets

The RNA-seq data of GM12878 were downloaded from NCBI SRA database (SRX457730) [38], and the reference transcriptome (version GRCh38.p13) of human was downloaded from NCBI Genome database. We used the same pipeline to identify the candidate editing sites in *H. sapiens* as described above. The candidate editing sites were further filtered using dbSNP 146 to remove the false editing sites.

### Nanopore direct RNA sequencing

#### *S. pombe* nanopore datasets

The Oxford Nanopore direct RNA sequencing was performed by Nextomics Bioscience (Wuhan, China). The sequencing libraries were constructed using ONT SQK-RNA001 kit following the manufacturer’s protocol (Oxford). The libraries were sequenced on GridION platform with R9.4 flowcells.

#### *H. sapiens* nanopore datasets

The nanopore direct RNA sequencing data on human cell line GM12878 were downloaded from GitHub (https://github.com/nanopore-wgs-consortium/NA12878). The direct RNA sequencing library was built using ONT SQK-RNA001 kit and sequenced on MinION or GridION using ONT R9.4 flowcells [22]. About 13 million nanopore reads were obtained and processed.

### Analysis of nanopore sequencing data

#### Basecalling and mapping

Nanopore reads were base-called using Guppy (version 4.0.15) with options –flowcell FLO-MIN106 –kit SQK-RNA001 – qscore_filtering –fast5_out. We employed Tombo [39] (version 1.5.1) to re-squiggle the raw electrical signals to reference transcriptome bases with option –include-event-stdev. Base-called fastq reads were filtered using NanoFilt [40] (version 2.7.1) with options -q 7 -l 150 to remove the low-quality and short reads. Minimap2 [41] (version 2.10-r761) was employed to map the filtered reads to the reference transcriptome (GCF_000002945.1_ASM294v2_rna.fna) with option –secondary = no. The mapped bam files were filtered using Samtools view (-F 4 -F 16 -F 2064) to remove the reads that were unmapped or mapped to the complementary strand. The bam files of biological replicates from the same *S. pombe* strain were merged for subsequent analysis.

#### KS-test of electrical signal values between FY-HFF1 and FY-ADAR2 samples

Sites with reads coverage ≥ 30 in both FY-HFF1 and FY-ADAR2 samples were retained. For each site, the norm_mean values of nanopore bases mapped on this site were extracted from FY-HFF1 and FY-ADAR2, and KS-test was performed between the two samples using the extracted signal values. We compared the *p*-values of editing sites and flanking regions (− 5 to + 5). Some editing sites were excluded due to the low read coverage in these regions. A total of 2318 editing sites were obtained, and 2318 unedited “A” sites randomly selected were used as control.

#### Identification of ADAR2-specific errors (ASEs)

To distinguish the random base-calling errors from base-calling errors due to RNA editing events (ADAR2-specific errors), we used the following criteria to select ADAR2-specific errors (ASE): (1) the SNV that owned the most support reads was selected as the base-calling error for each site; (2) the base-calling error occurred in FY-ADAR2 samples and not in FY-HFF1 samples, or the error ratio (number of error reads over the number of total reads) in FY-ADAR2 samples are at least twice that in FY-HFF1 samples; and (3) at least 5 nanopore reads support a base-calling error with error ratio ≥ 0.1.

#### Separation of I-type reads in FY-ADAR2 samples

We separated the nanopore reads mapped to editing sites in FY-ADAR2 samples that are ADAR2-specific errors (ASEs). The nanopore reads harboring ASEs around editing sites were annotated as I-type reads and the remaining reads were annotated as A-type reads (Additional file 1: Fig. S3b). We first selected the editing sites that own A-to-G ASEs in − 1 position (the editing sites were defined as 0 position), A-to-G ASEs in 0 position, and A-to-G and C-to-T ASEs in + 1 position. To avoid the signal perturbation of context bases, the editing sites that are located adjacent to other editing sites in a range of three nucleotide bases were excluded from consideration.

#### Feature extraction

Features of I-type reads were extracted as positive control and those of HFF1 reads from FY-HFF1 were used as negative control. Four features, including norm_mean, norm_std, length, and base identity for the six bases around the editing sites in I-type reads and HFF1 reads were extracted. Note the values of feature “length” were normalized by dividing by 100 to facilitate training. The detailed feature files are available at https://github.com/weir12/DeepEdit. About 80% of positive and negative control data were used for training, while the remaining data were used for testing.

### Training and testing of DeepEdit

#### Framework of DeepEdit

The core of DeepEdit is a fully connected neural network model, which consists of one input layer, two hidden layers, and one output layer (Fig. 2a). There are 1024 neurons in the input layer, 512 neurons in the first hidden layer, 64 neurons in the second hidden layer, and one neuron in the output layer. The output layer is activated using softmax activation function, and the output is the probability to be a positive sample. We regularized the neural network using dropout to avoid overfitting.

#### Evaluation of DeepEdit on human data

We selected completely edited and unedited sites to evaluate the performance of DeepEdit. An editing site was considered to be completely edited if its editing ratio is over 0.9; meanwhile, a site with an editing ratio = 0 was considered to be unedited. The nanopore reads mapped on completely edited sites were labeled as positive control and the ones mapped on unedited sites were labeled as negative control. The extracted features of selected nanopore reads were used as input. DeepEdit generated the editing probability for each nanopore read. The prediction data were compared with the known labels for calculating the AUC scores. When evaluating partially edited sites (editing ratio ≥ 0.1) and unedited sites (editing ratio = 0), the sites with mapped nanopore reads less than 30 were abandoned. A nanopore read was considered to be edited when having a predicted probability ≥ 0.9. The editing ratio of each site was calculated by counting the number of total mapped reads and that of predicted edited reads. When comparing the overlap of the detected editing sites between studies, we collected two atlases of RNA editing sites that were reported by previously published studies and conducted comparisons with the sites detected by our approach. From the first study by Ramaswami et al. [23], a total of 598 RNA editing sites, with a read depth of at least 5 in our nanopore data, were selected for evaluation (Additional file 2: Table S4). The sites with a predicted editing ratio ≥ 0.1 were regarded to be edited in our nanopore datasets. DeepEdit identified 338 of these sites (56.52%). From another study by Peng et al. [24], we evaluated 311 editing sites and found that 171 sites (54.98%) were identified as editing sites in our nanopore datasets (Additional file 2: Table S5).

## Supplementary Information

**Additional file 1:** **Fig. S1.** Implementation of hADAR2 editing system in S. pombe. **Fig. S2.** Confirmation of candidate A-to-I RNA editing sites by sanger sequencing. **Fig. S3.** Base-calling errors can be used to identify I-type reads in direct Nanopore reads. **Fig. S4.** Performance of models with different feature selection. **Fig. S5.** Challenge of reads mapping in repetitive elements by the short-read sequencing. **Fig. S6.** Changes in potential hydrogen bond interactions between bases induced by A-to-I RNA editing events in transcript NM_002794.5.

**Additional file 2:** **Table S1.** Number of ADAR2-specific errors (ASEs) around editing sites in *S.pombe*. **Table S2.** Number of ASEs around random ‘A’ sites in *S.pombe*. **Table S3.** Primers used in experiments. **Table S4. **Predicted editing levels of sites collected from Ramaswami et al. **Table S5. **Predicted editing levels of sites collected from Peng et al. **Table S6. **Detected editing sites in Alu regions of 10 human transcripts.

**Additional file 3.** Review history.

## Data Availability

All sequencing data generated in this study have been deposited in the European Nucleotide Archive (ENA) at EMBL-EBI and are publicly accessible under accession number PRJEB46364 (https://www.ebi.ac.uk/ena/browser/view/PRJEB46364) [42]. The raw nanopore direct RNA sequencing data of human used in this study is downloaded from GitHub (https://github.com/nanopore-wgs-consortium/NA12878) [22]. The Illumina RNA-seq data of human is downloaded from Sequence Read Archive (SRA) under accession number SRX457730 [38]. DeepEdit is publicly available under the MIT license at GitHub (https://github.com/weir12/DeepEdit) [43] and Zenodo (https://www.zenodo.org/record/7615493) [44].

## References

[1] Benne R, Van Den Burg J, Brakenhoff JPJ, Sloof P, Van Boom JH, Tromp MC (1986). Major transcript of the frameshifted coxll gene from trypanosome mitochondria contains four nucleotides that are not encoded in the DNA. Cell.

[2] Powell LM, Wallis SC, Pease RJ, Edwards YH, Knott TJ, Scott J (1987). A novel form of tissue-specific RNA processing produces apolipoprotein-B48 in intestine. Cell.

[3] Yu Y, Zhou H, Kong Y, Pan B, Chen L, Wang H, Hao P, Li X (2016). The landscape of A-to-I RNA editome is shaped by both positive and purifying selection. PLoS Genet.

[4] Nishikura K (2010). Functions and regulation of RNA editing by ADAR deaminases. Annu Rev Biochem.

[5] Bahn JH, Lee JH, Li G, Greer C, Peng G, Xiao X (2012). Accurate identification of A-to-I RNA editing in human by transcriptome sequencing. Genome Res.

[6] Park E, Williams B, Wold BJ, Mortazavi A (2012). RNA editing in the human ENCODE RNA-seq data. Genome Res.

[7] Bass BL, Nishikura K, Keller W, Seeburg PH, Emeson RB, O'Connell MA, Samuel CE, Herbert A (1997). A standardized nomenclature for adenosine deaminases that act on RNA. RNA.

[8] Bass BL, Weintraub H (1988). An unwinding activity that covalently modifies its double-stranded-RNA substrate. Cell.

[9] Wagner RW, Smith JE, Cooperman BS, Nishikura K (1989). A double-stranded RNA unwinding activity introduces structural alterations by means of adenosine to inosine conversions in mammalian cells and Xenopus eggs. Proc Natl Acad Sci USA.

[10] Bass BL (2002). RNA editing by adenosine deaminases that act on RNA. Annu Rev Biochem.

[11] Rueter SM, Dawson TR, Emeson RB (1999). Regulation of alternative splicing by RNA editing. Nature.

[12] Lev-Maor G, Sorek R, Levanon EY, Paz N, Eisenberg E, Ast G (2007). RNA-editing-mediated exon evolution. Genome Biol.

[13] Garalde DR, Snell EA, Jachimowicz D, Sipos B, Lloyd JH, Bruce M, Pantic N, Admassu T, James P, Warland A (2018). Highly parallel direct RNA sequencing on an array of nanopores. Nat Methods.

[14] Liu H, Begik O, Lucas MC, Ramirez JM, Mason CE, Wiener D, Schwartz S, Mattick JS, Smith MA, Novoa EM (2019). Accurate detection of m(6)A RNA modifications in native RNA sequences. Nat Commun.

[15] Parker MT, Knop K, Sherwood AV, Schurch NJ, Mackinnon K, Gould PD, Hall AJ, Barton GJ, Simpson GG: Nanopore direct RNA sequencing maps the complexity of Arabidopsis mRNA processing and m(6)A modification. Elife. 2020;9:e49658. 10.7554/eLife.49658 PMC6959997 31931956

[16] Gao Y, Liu X, Wu B, Wang H, Xi F, Kohnen MV, Reddy ASN, Gu L (2021). Quantitative profiling of N(6)-methyladenosine at single-base resolution in stem-differentiating xylem of Populus trichocarpa using Nanopore direct RNA sequencing. Genome Biol.

[17] Qin H, Ou L, Gao J, Chen L, Wang J-W, Hao P, Li X (2022). DENA: training an authentic neural network model using Nanopore sequencing data of Arabidopsis transcripts for detection and quantification of N6-methyladenosine on RNA. Genome Biol.

[18] Viehweger A, Krautwurst S, Lamkiewicz K, Madhugiri R, Ziebuhr J, Hölzer M, Marz M (2019). Direct RNA nanopore sequencing of full-length coronavirus genomes provides novel insights into structural variants and enables modification analysis. Genome Res.

[19] Begik O, Lucas MC, Pryszcz LP, Ramirez JM, Medina R, Milenkovic I, Cruciani S, Liu H, Vieira HGS, Sas-Chen A (2021). Quantitative profiling of pseudouridylation dynamics in native RNAs with nanopore sequencing. Nat Biotechnol.

[20] Xu L, Seki M (2020). Recent advances in the detection of base modifications using the Nanopore sequencer. J Hum Genet.

[21] Nguyen TA, Heng JWJ, Kaewsapsak P, Kok EPL, Stanojević D, Liu H, Cardilla A, Praditya A, Yi Z, Lin M (2022). Direct identification of A-to-I editing sites with nanopore native RNA sequencing. Nat Methods.

[22] Workman RE, Tang AD, Tang PS, Jain M, Tyson JR, Razaghi R, Zuzarte PC, Gilpatrick T, Payne A, Quick J (2019). Nanopore native RNA sequencing of a human poly(A) transcriptome. Nat Methods.

[23] Ramaswami G, Lin W, Piskol R, Tan MH, Davis C, Li JB (2012). Accurate identification of human Alu and non-Alu RNA editing sites. Nat Methods.

[24] Peng Z, Cheng Y, Tan BC-M, Kang L, Tian Z, Zhu Y, Zhang W, Liang Y, Hu X, Tan X (2012). Comprehensive analysis of RNA-Seq data reveals extensive RNA editing in a human transcriptome. Nat Biotechnol.

[25] Li JB, Levanon EY, Yoon J-K, Aach J, Xie B, LeProust E, Zhang K, Gao Y, Church GM (2009). Genome-wide identification of human RNA editing sites by parallel DNA capturing and sequencing. Science (80-).

[26] Vollmar W, Gloger J, Berger E, Kortenbruck G, Köhling R, Speckmann EJ, Musshoff U (2004). RNA editing (R/G site) and flip-flop splicing of the AMPA receptor subunit GluR2 in nervous tissue of epilepsy patients. Neurobiol Dis.

[27] Gaisler-Salomon I, Kravitz E, Feiler Y, Safran M, Biegon A, Amariglio N, Rechavi G (2014). Hippocampus-specific deficiency in RNA editing of GluA2 in Alzheimer's disease. Neurobiol Aging.

[28] Khermesh K, D'Erchia AM, Barak M, Annese A, Wachtel C, Levanon EY, Picardi E, Eisenberg E (2016). Reduced levels of protein recoding by A-to-I RNA editing in Alzheimer's disease. RNA.

[29] Baker AR, Slack FJ (2022). ADAR1 and its implications in cancer development and treatment. Trends Genet.

[30] Gumireddy K, Li A, Kossenkov AV, Sakurai M, Yan J, Li Y, Xu H, Wang J, Zhang PJ, Zhang L (2016). The mRNA-edited form of GABRA3 suppresses GABRA3-mediated Akt activation and breast cancer metastasis. Nat Commun.

[31] Jing X, Xie B, Chen L, Zhang N, Jiang Y, Qin H, Wang H, Hao P, Yang S, Li X (2018). Implementation of the CRISPR-Cas13a system in fission yeast and its repurposing for precise RNA editing. Nucleic Acids Res.

[32] Matsuyama A, Yoshida M (2012). Heterologous gene expression by chromosomal integration in fission yeast. Methods Mol Biol.

[33] Sabatinos SA, Forsburg SL (2010). Molecular genetics of Schizosaccharomyces pombe. Meth Enzymol.

[34] Xu H, Luo X, Qian J, Pang X, Song J, Qian G, Chen J, Chen S (2012). FastUniq: a fast de novo duplicates removal tool for paired short reads. PLoS ONE.

[35] Kim D, Paggi JM, Park C, Bennett C, Salzberg SL (2019). Graph-based genome alignment and genotyping with HISAT2 and HISAT-genotype. Nat Biotechnol.

[36] Li H, Handsaker B, Wysoker A, Fennell T, Ruan J, Homer N, Marth G, Abecasis G, Durbin R, Genome Project Data Processing S (2009). The sequence alignment/map format and SAMtools. Bioinformatics.

[37] Li H (2011). A statistical framework for SNP calling, mutation discovery, association mapping and population genetical parameter estimation from sequencing data. Bioinformatics.

[38] Tilgner H, Grubert F, Sharon D, Snyder MP (2014). Defining a personal, allele-specific, and single-molecule long-read transcriptome. Proc Natl Acad Sci USA.

[39] De novo identification of DNA modifications enabled by genome-guided nanopore signal processing. bioRxiv. 2017:094672. 10.1101/094672.

[40] De Coster W, D'Hert S, Schultz DT, Cruts M, Van Broeckhoven C (2018). NanoPack: visualizing and processing long-read sequencing data. Bioinformatics.

[41] Li H (2018). Minimap2: pairwise alignment for nucleotide sequences. Bioinformatics.

[42] Chen L, Ou L, Jing X, Kong Y, Xie B, Zhang N, Shi H, Qin H, Li X, Hao P. DeepEdit: single-molecule detection and phasing of A-to-I RNA editing events using Nanopore direct RNA sequencing. Datasets. European Nucleotide Archive. https://www.ebi.ac.uk/ena/browser/view/PRJEB46364 (2023). 10.1186/s13059-023-02921-0 PMC10108526 37069604

[43] Chen L, Ou L, Jing X, Kong Y, Xie B, Zhang N, Shi H, Qin H, Li X, Hao P. DeepEdit: single-molecule detection and phasing of A-to-I RNA editing events using Nanopore direct RNA sequencing. Github. https://github.com/weir12/DeepEdit (2023). 10.1186/s13059-023-02921-0 PMC10108526 37069604

[44] Chen L, Ou L, Jing X, Kong Y, Xie B, Zhang N, Shi H, Qin H, Li X, Hao P. DeepEdit: single-molecule detection and phasing of A-to-I RNA editing events using Nanopore direct RNA sequencing. Zenodo. https://www.zenodo.org/record/7615493 (2023). 10.1186/s13059-023-02921-0 PMC10108526 37069604

