# Supplementary material for: DeepEdit: single-molecule detection and phasing of A-to-I RNA editing events using nanopore direct RNA sequencing
Source: Genome Biol. 2023 Apr 17;24:75. doi: 10.1186/s13059-023-02921-0 (PMC10108526; doi:10.1186/s13059-023-02921-0)
Supplement: Supplementary file 1 — Additional file 1: Fig. S1. Implementation of hADAR2 editing system in S. pombe. Fig. S2. Confirmation of candidate A-to-I RNA editing sites by sanger sequencing. Fig. S3. Base-calling errors can be used to identify I-type reads in direct Nanopore reads. Fig. S4. Performance of models with different feature selection. Fig. S5. Challenge of reads mapping in repetitive elements by the short-read sequencing. Fig. S6. Changes in potential hydrogen bond interactions between bases induced by A-to-I RNA editing events in transcript NM_002794.5. [file 13059_2023_2921_MOESM1_ESM.docx]

Additional file1

**DeepEdit: single-molecule detection and phasing of A-to-I RNA editing events using Nanopore direct RNA sequencing**

Longxian Chen^1,†^, Liang Ou^2,†^, Xinyun Jing^1,†^, Yimeng Kong^3,†^, Bingran Xie^1^, Niubing Zhang^1^, Han Shi^1,4^, Hang Qin^1^, Xuan Li^1,4,^*, and Pei Hao^2,4,^*

^1^Key Laboratory of Synthetic Biology, CAS Center for Excellence in Molecular Plant Sciences, Institute of Plant Physiology and Ecology, Chinese Academy of Sciences, Shanghai, China

^2^Key Laboratory of Molecular Virology and Immunology, Institut Pasteur of Shanghai, Chinese Academy of Sciences, Shanghai, China

^3^Department of Genetics and Genomic Sciences and Icahn Institute for Genomics and Multiscale Biology, Icahn School of Medicine at Mount Sinai, New York, NY, USA

^4^University of Chinese Academy of Sciences, Beijing, China

* Corresponding author

† These authors contributed equally to this work

Xuan Li: lixuan@sippe.ac.cn

Pei Hao: phao@ips.ac.cn

**Contents**

**Fig. S1** Implementation of hADAR2 editing system in S. pombe.

**Fig. S2** Confirmation of candidate A-to-I RNA editing sites by sanger sequencing.

**Fig. S3** Base-calling errors can be used to identify I-type reads in direct Nanopore reads.

**Fig. S4** Performance of models with different feature selection.

**Fig. S5** Challenge of reads mapping in repetitive elements by the short-read sequencing.

**Fig. S6** Changes in potential hydrogen bond interactions between bases induced by A-to-I RNA editing events in transcript NM_002794.5.

**Fig. S1**

**
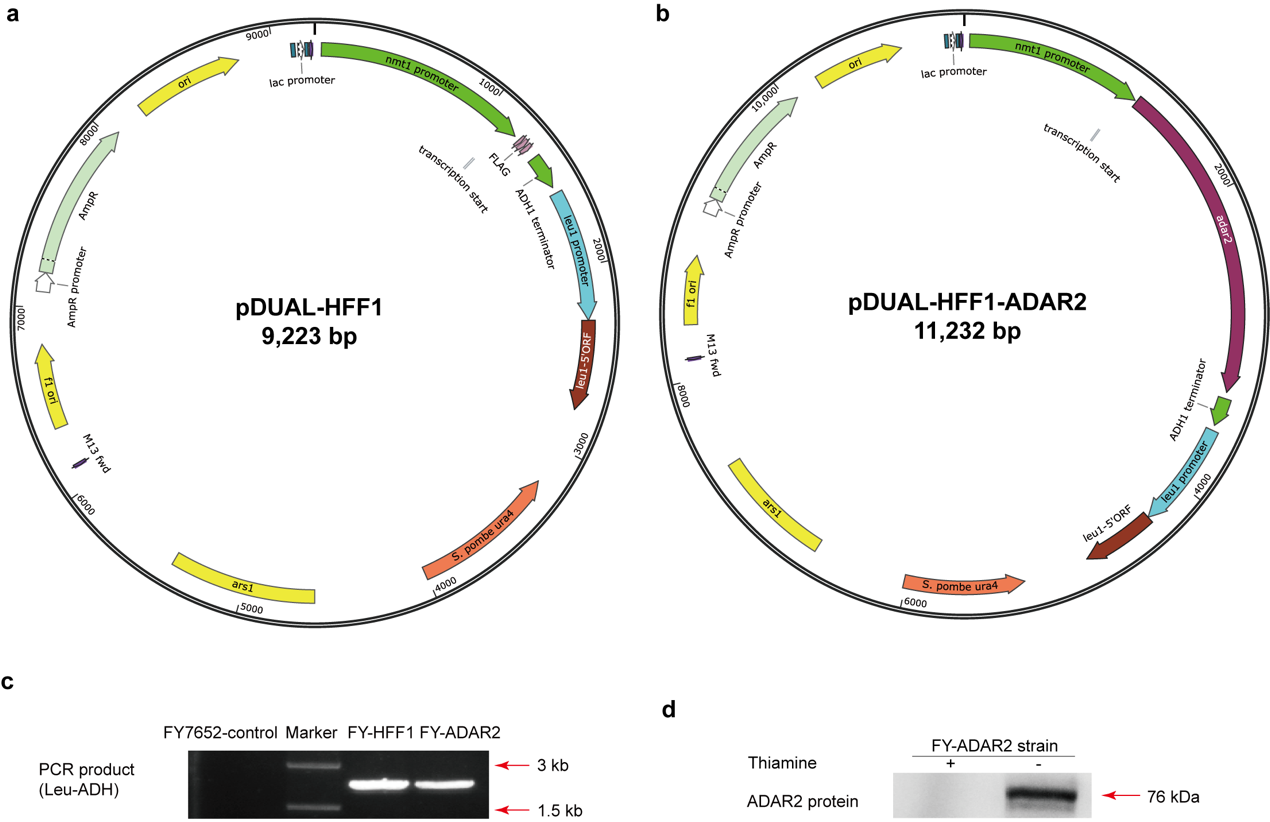
**

**Fig. S1 Implementation of hADAR2 editing system in S. pombe.**

**a, b** An *Escherichia coli*–*Schizosaccharomyces pombe* shuttle plasmid (pDUAL- HFF1)**(a)** was modified to carry the hADAR2 expression cassette **(b)**.

**c** PCR and gel-electrophoresis validation of hADAR2 coding sequence insertion.

**d** Western blotting of hADAR2 protein. “+” means the culture contains thiamine in it and “-” means not.

**Fig. S2**

**
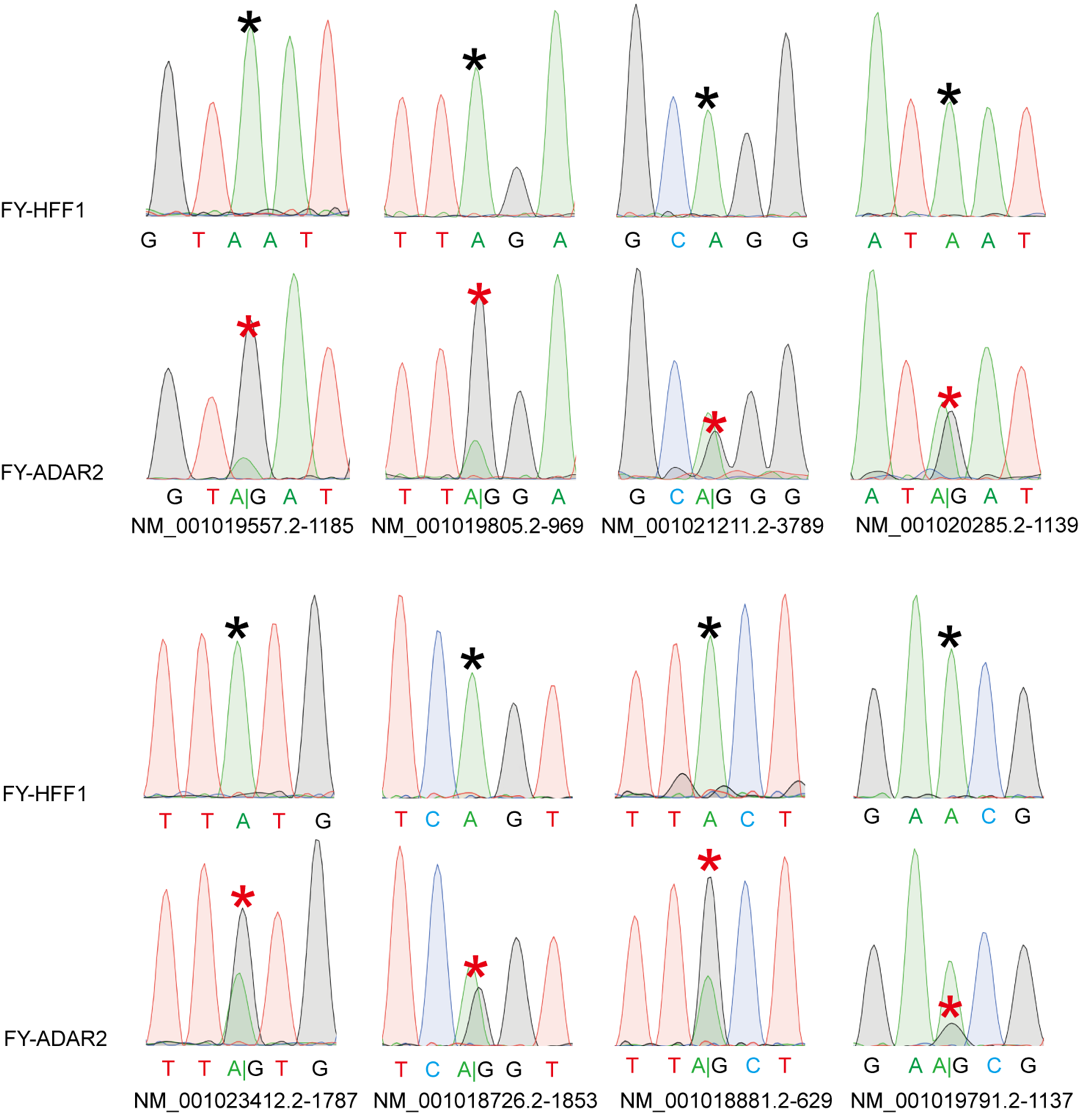
**

**Fig. S2 Confirmation of candidate A-to-I RNA editing sites by sanger sequencing.** Red asterisks denote the detected editing events, and black asterisks indicate that no editing events were detected.

**Fig. S3**


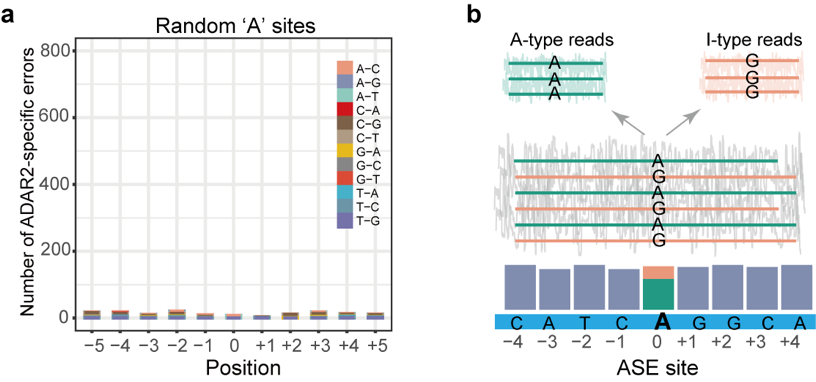


**Fig. S3 Base-calling errors can be used to identify I-type reads in direct Nanopore reads.**

**a** Frequencies of different types of ADAR2-specific errors (ASEs) around random ‘A’ sites, shown with bases from -5 to +5 around editing sites.

**b** Workflow of read-separation using ASEs as benchmarks. Bases from -4 to +4 surrounding the editing sites were shown with the correct reference bases shown on the bottom. The gray bars denote the bases on Nanopore reads are same as reference bases. The colored bars denote the occurrence of ASEs between Nanopore reads and reference. The Nanopore reads owning a mismatch on ASE site were classified as I-type reads (edited reads), meanwhile the reads owning the same bases as reference were classified as A-type reads (unedited reads).

**Fig. S4**

**
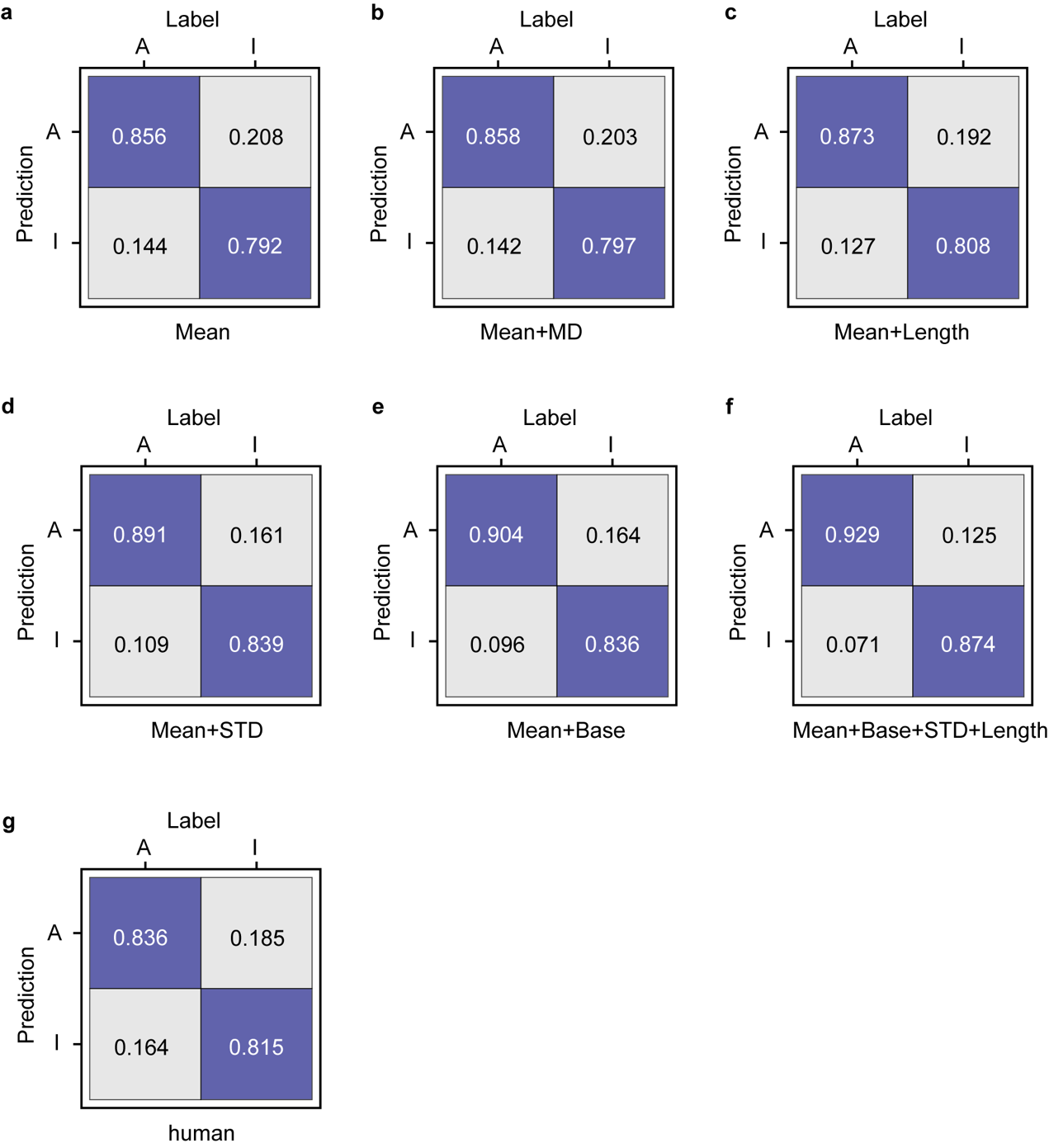
**

**Fig. S4 Performance of models with different feature selection.** In *S. pombe,* selected feature combinations, including Mean **(a)**, Mean and MD **(b)**, Mean and Length **(c)**, Mean and STD **(d)**, Mean and Base **(e)**, and combination of all the above features except MD**(f)** were tested to get the best performance. Mean, normalized electrical signal means. MD, mean deviations between adjacent bases. STD, standard deviations. Length, the number of raw signal values. Base, base type of ‘A’, ‘T’, ‘C’, ‘G’. **g** The performance of DeepEdit on human datasets. Confusion matrices for each test were shown, with rows denote the actual bases and columns denote perditions by the model.

**Fig. S5**


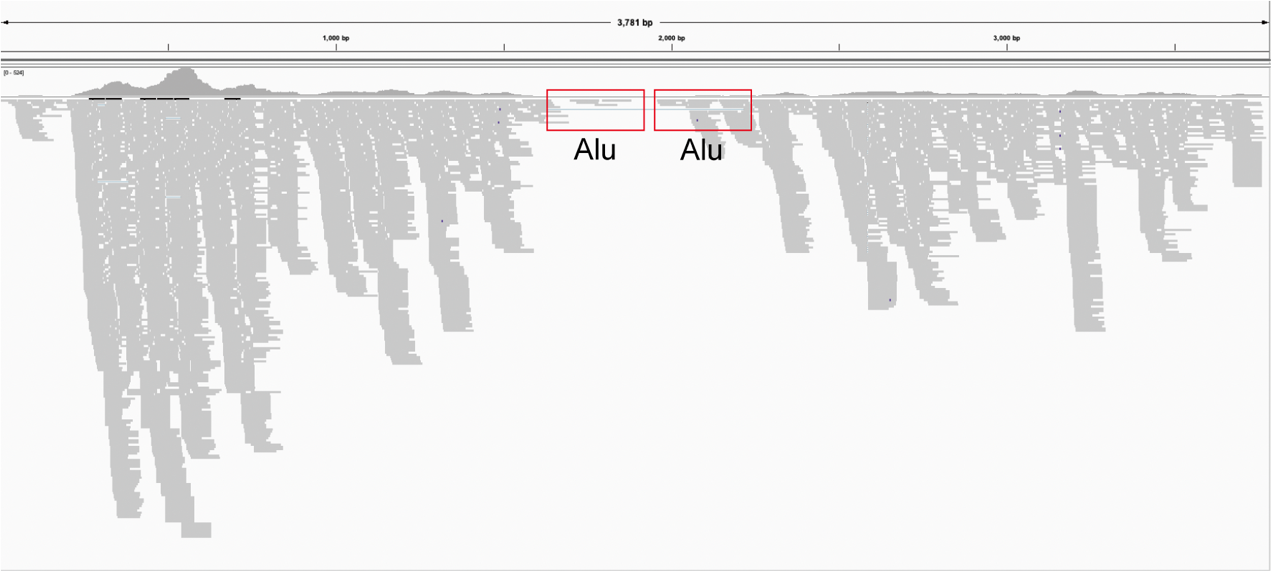


**Fig. S5 Challenge of reads mapping in repetitive elements by the short-read sequencing.** As illustrated by the snapshot of the Integrative Genomics Viewer (IGV), the depth of coverage in Alu repeat regions (indicated by red boxes) is significantly lower compared to other regions on transcript NM_015704.3. This reduction in depth of coverage highlights the difficulties in accurately mapping short reads in repetitive elements, which can lead to an underestimation of the number of editing sites.

**Fig. S6**


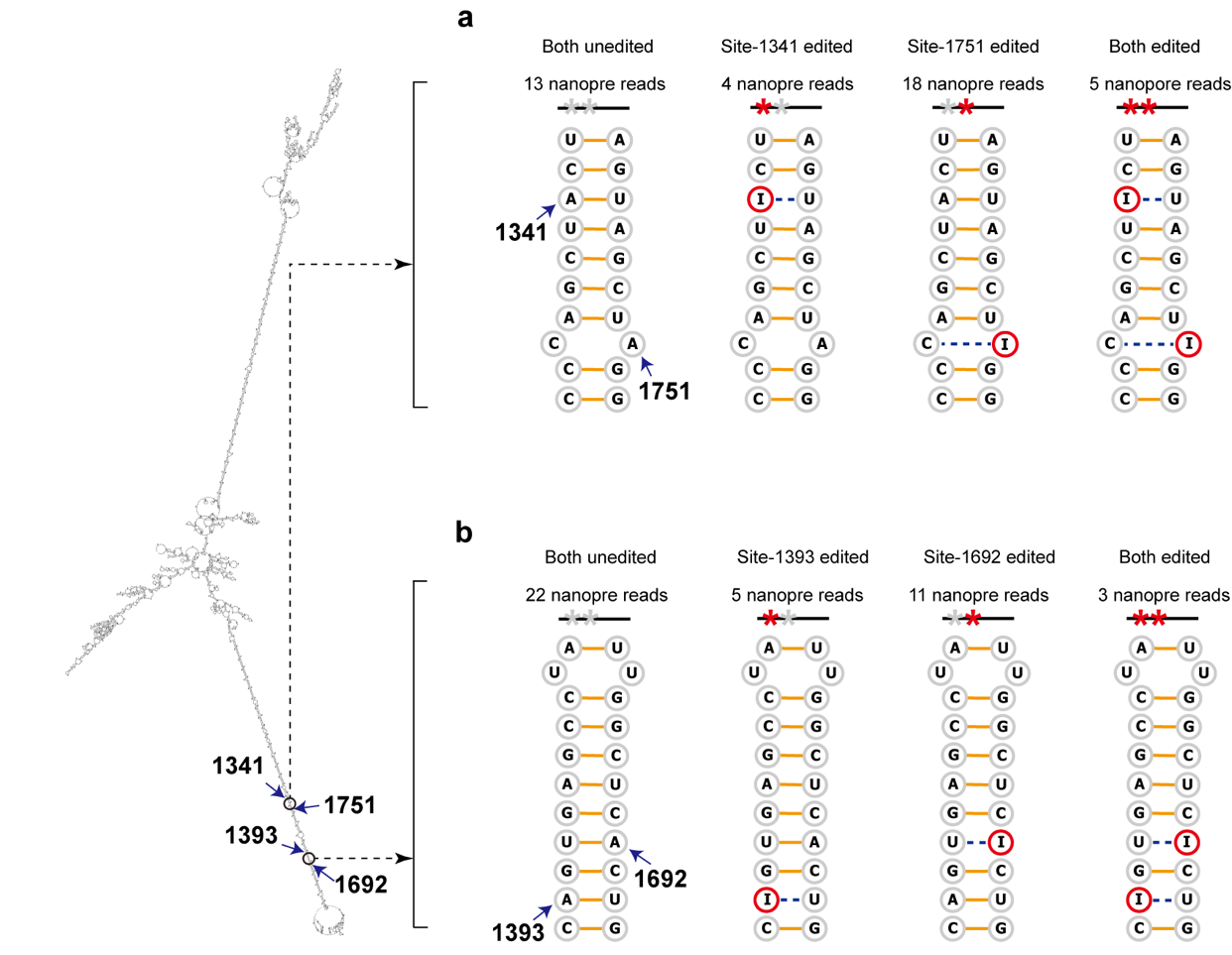


**Fig. S6 Changes in potential hydrogen bond interactions between bases induced by A-to-I RNA editing events in transcript NM_002794.5.** The predicted secondary structure of the RNA transcript is shown on the left, with enlarged views of secondary structures at sites 1,341/1,751 (**a**) and 1,393/1,692 (**b**). Edited and unedited sites on reads are denoted by red and gray asterisks, respectively. The orange lines represent the predicted base pairing between nucleotides using RNAfold. Dashed lines indicate disrupted or newly formed hydrogen bonds resulting from A-to-I editing events.
