# Supplementary material for: DeepEdit: single-molecule detection and phasing of A-to-I RNA editing events using nanopore direct RNA sequencing
Source: Genome Biol. 2023 Apr 17;24:75. doi: 10.1186/s13059-023-02921-0 (PMC10108526; doi:10.1186/s13059-023-02921-0)
Supplement: Supplementary file 3 — Additional file 3. Review history. [file 13059_2023_2921_MOESM3_ESM.docx]

Review History

**First round of review**

**Reviewer 1**

**Were you able to assess all statistics in the manuscript, including the appropriateness of statistical tests used?** There are no statistics in the manuscript.

**Comments to author:**

In this manuscript, the authors developed a neural network model, DeepEdit, that recognizes A-to-I editing events in single reads of Oxford Nanopore direct RNA sequencing, and resolves the phasing of RNA editing events on transcripts. The topic in important, and the manuscript is well written and organized. The reviewer has several comments:

1. For human data, could the authors compare the performance of long reads vs. short reads in detecting RNA editing sites? For example, if using long reads can identify more RNA editing sites?

2. The potential application of the pipeline in human diseases, e.g., cancer may be discussed.

3. The data PRJNA863035 seems cannot be accessed.

**Reviewer 2**

**Were you able to assess all statistics in the manuscript, including the appropriateness of statistical tests used?** No, I do not feel adequately qualified to assess the statistics.

**Comments to author:**

The manuscript "Single-molecule detecting and phasing of A-to-I RNA editing events using nanopore direct RNA sequencing " by Chen et al. deals with the identification of A-to-I RNA editing events directly by Oxford sequencing technology

In the last few years, several papers were published with the aim of identifying DNA and RNA modification directly by sequencing, utilizing nanopore sequencing. This is of high importance topic as it can reveal the occurrence of the modification by single base resolution and allow the study of their functional significance. Out of the many modifications known so far, A-to-I RNA editing may be the only modification that some may claim that the use of such an approach is not a necessity. This is because A-to-I RNA editing, unlike all other modifications, can be identified directly from Illumina sequencing. Yet, even for A-to-I RNA editing, having the ability to measure editing in along reads has some important applications, namely the ability to get haplotypes.

In this paper, the authors did a nice job extracting editing events from Nanopore technology and developing a computational tool to do so. Yet several issues should be addressed before publication:

1) It needs to be clarified how and if, this approach is better than previously published methods. The authors should elaborate more on this point.

2) A-to-I RNA editing occurs in dsRNA structures. Thus, the main advantage of having long reads is the ability to find the impact of sites that are close on the structure, even if they are a long distance on the sequence. -some such examples should be presented

3) Atlases of RNA editing events were published in the last few years- it should be essential to demonstrate the overlap in the sites that were detected and the ones that were published, in order to evaluate the validity of the approach.

**Authors Response**

**Point-by-point Responses to Reviewers’ Comments**

**SUBMISSION:** GBIO-D-22-01640

**TITLE:** Single-molecule detection and phasing of A-to-I RNA editing events using nanopore direct RNA sequencing

**AUTHORS:** Longxian Chen; Liang Ou; Xinyun Jing; Yimeng Kong; Bingran Xie; Niubing Zhang; Han Shi; Hang Qin; Xuan Li; Pei Hao

We highly appreciate the insightful and constructive comments from the reviewers. According to the reviewers’ comments, we have extensively revised the manuscript to address all the questions and provided point-by-point responses. Responses to reviewers’ comments are in blue text and cited references are included at the end.

**Reviewer reports:**

**Reviewer #1:** In this manuscript, the authors developed a neural network model, DeepEdit, that recognizes A-to-I editing events in single reads of Oxford Nanopore direct RNA sequencing and resolves the phasing of RNA editing events on transcripts. The topic in important, and the manuscript is well written and organized. The reviewer has several comments:

We thank this referee very much for the positive comments and thorough review of our work. In the revised manuscript, we have addressed all the comments and summarized new data and discussions suggested by the reviewer.

1. For human data, could the authors compare the performance of long reads vs. short reads in detecting RNA editing sites? For example, if using long reads can identify more RNA editing sites?

Response:

We thank the reviewer for this great suggestion. Our long-read method can identify more RNA editing events compared to the short-read approaches (NGS). This is due to the high proportion of repetitive elements in mammalian genomes. For example, Repeatmasker has identified that more than 50% of the human genome is marked by repeat elements[1]. These repeat elements can only be accurately detected with long reads, as they are more likely to be misaligned or unaligned by short-read data[2]. Additionally, regions with high levels of RNA editing, known as hyper-edited regions, may also be challenging for accurate short-read mapping due to the presence of abundant A-to-G mismatches[3].

In our previous manuscript, we demonstrated that our long-read method, DeepEdit, exhibits comparable performance with the canonical short-read method (NGS) (Fig.2c, d). Both long-read and short-read sequencing methods can accurately identify editing sites in non-repetitive and moderately edited regions.

However, for the repetitive elements and hyper-edited regions, our method DeepEdit has the advantage of detecting more editing sites. As an example, we screened the Alu repeat regions on 10 transcripts and detected 104 editing sites (Additional file2: Table S6), 96 sites (92.30%) of which were annotated as potential editing sites in the REDIportal database (An ATLAS of A-to-I RNA editing events in humans)[4]. In contrast, only 50 sites were detected using the short-read method. For instance, the transcript NM_015704.3 contains two Alu repeat elements, where DeepEdit was able to identify 8 editing sites in the Alu repeat regions, all of which were annotated as edited in the REDIportal database. Conversely, the short-read approach failed to detect these editing sites, due to the difficulty of short-read alignment in these regions (Additional file1: Fig. S5).

Therefore, compared to short-read methods, the use of long reads in RNA editing detection can indeed identify more editing sites in hyper-edited or repeated regions. Many of the repetitive elements have been linked to human disease[5], emphasizing the distinct advantage of using long-read sequencing for RNA-editing detection. We have added these important points in the revised manuscript (page 8, lines 177-184).

**Additional file1: Fig. S5. Challenge of reads mapping in Alu regions by the short-read sequencing.** As illustrated by the snapshot of the Integrative Genomics Viewer (IGV), the depth of coverage in Alu repeat regions (indicated by red boxes) is significantly lower compared to other regions on transcript NM_015704.3. This reduction in depth of coverage highlights the difficulties in accurately mapping short reads in repetitive elements and hyper-edited regions, which can lead to an underestimation of the number of editing sites.

2. The potential application of the pipeline in human diseases, e.g., cancer may be discussed.

Response:

We thank the reviewer for the comment. Indeed, RNA editing plays important roles in a growing list of diseases, including neurological disorders[6-8] and cancers[9, 10], via manipulating variable molecular processes such as protein re-coding and transcript splicing. Although the short-read method has been widely used in RNA editing research, it is still challenging to estimate the influence of RNA editing on protein re-coding or splicing in some cases. One prominent example of this case is the serotonin receptor HTR2C, which showed a reduced level of A-to-I RNA editing in Alzheimer’s disease[8]. There are five potential editing sites on a certain transcript, which were not fully edited or unedited, and potentially lead to 24 different protein isoforms. The short-read method is unable to estimate the possible protein isoforms in such cases if the editing sites span a long distance. However, with the benefit of long reads and single-molecule resolution, DeepEdit could identify the editing events on each transcript isoform, and thus directly reveal the potential protein products (Fig. 2f). DeepEdit could also exert more on splicing research. The RNA editing sites could create or destroy new splice events in cancers. By identifying the editing and splicing events on each transcript molecule, we could estimate the influence of RNA editing on splicing events more directly.

Due to word limitation, we combined the discussions on the unique advantages of our method and potential applications in human diseases in one paragraph of our revised manuscript. Please refer to the revised manuscript for detailed information (page 9, lines 192-204).

3. The data PRJNA863035 seems cannot be accessed.

Response:

Thanks for the reviewer’s reminder. The sequencing data used in this study have been deposited in the European Nucleotide Archive (ENA) at EMBL-EBI and are publicly accessible under accession number PRJEB46364 (https://www.ebi.ac.uk/ena/browser/view/PRJEB46364).

**Reviewer #2:** The manuscript "Single-molecule detecting and phasing of A-to-I RNA editing events using nanopore direct RNA sequencing " by Chen et al. deals with the identification of A-to-I RNA editing events directly by Oxford sequencing technology.

In the last few years, several papers were published with the aim of identifying DNA and RNA modification directly by sequencing, utilizing nanopore sequencing. This is of high importance topic as it can reveal the occurrence of the modification by single base resolution and allow the study of their functional significance. Out of the many modifications known so far, A-to-I RNA editing may be the only modification that some may claim that the use of such an approach is not a necessity. This is because A-to-I RNA editing, unlike all other modifications, can be identified directly from Illumina sequencing. Yet, even for A-to-I RNA editing, having the ability to measure editing in along reads has some important applications, namely the ability to get haplotypes.

In this paper, the authors did a nice job extracting editing events from Nanopore technology and developing a computational tool to do so. Yet several issues should be addressed before publication:

We thank the reviewer very much for the encouraging positive feedback on our study. We agree with the reviewer’s comments on the advantage of nanopore sequencing for A-to-I editing phasing toward getting a haplotype, which was the primary goal of this study. In the revised manuscript, we have thoroughly addressed all the comments and incorporated new data and discussions as suggested by the reviewer.

1. It needs to be clarified how and if, this approach is better than previously published methods. The authors should elaborate more on this point.

Response:

Thanks for the reviewer’s comment. DeepEdit boasts several advantages over the previously published nanopore-based method, as it incorporates new functions developed in our study. We elaborated the comparison of our method, DeepEdit, with other methods in several key areas:

**1) Single-molecule detection capability**. Our method DeepEdit is currently the first method capable of single-molecule detection of RNA editing events on nanopore reads. In contrast, the published nanopore-based method (Dinopore), can only provide site-level editing results by aggregating reads at specific genomic locations [11]. The ability to perform single-molecule detection provides a significant advantage for DeepEdit.

**(2) Detection of editing sites independent of editing ratios.** DeepEdit is capable to identify the editing events independent of their editing ratios taking advantage of its single-molecule resolution. As demonstrated in the revised manuscript (page8, line175-179), DeepEdit can accurately predict RNA-editing events in Alu elements, which are typically edited with low ratios[12]. In contrast, previous methods such as Dinopore are unreliable in detecting lowly edited sites (e.g., < 10%).

**(3) Phasing of RNA-editing events.** As the reviewer mentioned, the use of long reads by nanopore enables the application of RNA-editing phasing and the determination of haplotypes. DeepEdit, with its single-molecule resolution, provides the ability to perform read-level prediction and obtain haplotype phasing information, which is not possible with the previously published nanopore-based method for lacking single-molecule analysis capabilities.

In conclusion, DeepEdit represents a significant improvement over the previously published nanopore-base method. With its ability to perform single-molecule editing analysis, accurately predict lower-editing sites, and phase RNA-editing events using its long reads, DeepEdit provides a valuable tool for RNA editing detection. We have added in-depth discussions on these advantages in the revised manuscript (page 9, lines 205-213).

2. A-to-I RNA editing occurs in dsRNA structures. Thus, the main advantage of having long reads is the ability to find the impact of sites that are close on the structure, even if they are a long distance on the sequence. -some such examples should be presented.

Response:

We agree with the reviewer’s comment on the benefit of using long reads to identify A-to-I RNA editings occurring in dsRNA structures. By combining the long-read-sequencing with the single-molecule analysis, we were able to demonstrate the impact of RNA editings on the dsRNA structures at the reads level, which however, is not achievable with the conventional short-read methods. In the revised manuscript, we included two examples to demonstrate the utility of our approach.

In the first example, we investigated two RNA editing sites on NM_002794.5, site 1,341 and 1,751, which are separated by 410bp in the primary sequence, but are close on the secondary structure (Additional file1: Fig. S6a). By applying DeepEdit, we reproduced the four potential isoforms, including 5 RNA molecules edited on both sites, 4 and 18 RNA molecules edited solely on site-1,341 and site-1,751, respectively, and 13 molecules unedited on both sites. These variably edited isoforms may form distinct RNA secondary structures and therefore alter the molecular functions of RNA. Similarly, the second example showed the structures of variably edited isoforms on site-1,393 and site-1,692, which are far apart in primary sequences but close on the dsRNA structure (Additional file1: Fig. S6b). By providing these actual isoforms in the transcriptome, our approach could serve as a useful tool for investigating the relationships between RNA editing events and secondary structure changes, which may further affect their biological functions, such as miRNA targeting[13] or RNA stabilization[14]. We have added these findings in the revised manuscript (page 9, lines 202-204).

**Additional file1: Fig. S6. Changes in potential hydrogen bond interactions between bases induced by A-to-I RNA editing events in transcript NM_002794.5.** The predicted secondary structure of the RNA transcript is shown on the left, with enlarged views of secondary structures at sites 1,341/1,751 (**a**) and 1,393/1,692 (**b**). Edited and unedited sites on reads are denoted by red and gray asterisks, respectively. The orange lines represent the predicted base pairing between nucleotides using RNAfold. Dashed lines indicate disrupted or newly formed hydrogen bonds resulting from A-to-I editing events.

3. Atlases of RNA editing events were published in the last few years. It should be essential to demonstrate the overlap in the sites that were detected and the ones that were published, in order to evaluate the validity of the approach.

Response:

We thank the reviewer for the comment. We evaluated the validity of our approach in the following two aspects:

1) In our previous manuscript, we demonstrated a comparable performance between our nanopore-based method, DeepEdit, with the conventional short-read (NGS) method using a matched sample (Fig. 2c and 2d).

2) Following the reviewer’s suggestions, we collected two atlases of RNA editing sites that were reported by previously published studies and conducted comparisons with the sites detected by our approach. From the first study by Ramaswami et al.[15], a total of 598 RNA editing sites, with a read depth of at least 5 in our nanopore data, were selected for evaluation (Additional file2: Table S4). Our results showed that DeepEdit identified 338 of these sites (56.52%). In addition, from another study by Peng et al.[16], we evaluated 311 editing sites and found that 171 sites (54.98%) were detected in our nanopore datasets (Additional file2: Table S5). Considering previous studies have shown overlap rates of 48.87% between Ramaswami et al.[15] and Li et al.[17], or 56.48% between Ramaswami et al.[15] and Peng et al.[16], our results represent a comparable overlap rate with previous studies. It is worth mentioning that these overlap rates between studies are expected due to the highly variable RNA editing events on the human transcriptome, which is influenced by factors such as variation between cell types and tissues; and the varying editing statuses of wide-spread Alu elements in human genome which are randomly sampled with limited sequencing yield each time[18].

In summary, all these evaluations demonstrated the validity of our approach. We have added these results to the revised manuscript (page 8, lines 168-175).

Cited references:

1.	Nishimura D: RepeatMasker. *Biotech Software & Internet Report* 2000, 1:36-39.

2.	Bahlo M, Bennett MF, Degorski P, Tankard RM, Delatycki MB, Lockhart PJ: Recent advances in the detection of repeat expansions with short-read next-generation sequencing. *F1000Res* 2018, 7.

3.	Kim D, Paggi JM, Park C, Bennett C, Salzberg SL: Graph-based genome alignment and genotyping with HISAT2 and HISAT-genotype. *Nat Biotechnol* 2019, 37:907-915.

4.	Picardi E, D'Erchia AM, Lo Giudice C, Pesole G: REDIportal: a comprehensive database of A-to-I RNA editing events in humans. *Nucleic Acids Res* 2017, 45:D750-d757.

5.	Gallo A, Vukic D, Michalík D, O’connell MA, Keegan LP: ADAR RNA editing in human disease; more to it than meets the I. *Hum Genet* 2017, 136:1265-1278.

6.	Vollmar W, Gloger J, Berger E, Kortenbruck G, Köhling R, Speckmann EJ, Musshoff U: RNA editing (R/G site) and flip-flop splicing of the AMPA receptor subunit GluR2 in nervous tissue of epilepsy patients. *Neurobiol Dis* 2004, 15:371-379.

7.	Gaisler-Salomon I, Kravitz E, Feiler Y, Safran M, Biegon A, Amariglio N, Rechavi G: Hippocampus-specific deficiency in RNA editing of GluA2 in Alzheimer's disease. *Neurobiol Aging* 2014, 35:1785-1791.

8.	Khermesh K, D'Erchia AM, Barak M, Annese A, Wachtel C, Levanon EY, Picardi E, Eisenberg E: Reduced levels of protein recoding by A-to-I RNA editing in Alzheimer's disease. *RNA* 2016, 22:290-302.

9.	Baker AR, Slack FJ: ADAR1 and its implications in cancer development and treatment. *Trends Genet* 2022, 38:821-830.

10.	Gumireddy K, Li A, Kossenkov AV, Sakurai M, Yan J, Li Y, Xu H, Wang J, Zhang PJ, Zhang L, et al: The mRNA-edited form of GABRA3 suppresses GABRA3-mediated Akt activation and breast cancer metastasis. *Nat Commun* 2016, 7:10715.

11.	Nguyen TA, Heng JWJ, Kaewsapsak P, Kok EPL, Stanojević D, Liu H, Cardilla A, Praditya A, Yi Z, Lin M, et al: Direct identification of A-to-I editing sites with nanopore native RNA sequencing. *Nat Methods* 2022, 19:833-844.

12.	Bazak L, Haviv A, Barak M, Jacob-Hirsch J, Deng P, Zhang R, Isaacs FJ, Rechavi G, Li JB, Eisenberg E, Levanon EY: A-to-I RNA editing occurs at over a hundred million genomic sites, located in a majority of human genes. *Genome Res* 2014, 24:365-376.

13.	Brümmer A, Yang Y, Chan TW, Xiao X: Structure-mediated modulation of mRNA abundance by A-to-I editing. *Nat Commun* 2017, 8:1255.

14.	Stellos K, Gatsiou A, Stamatelopoulos K, Perisic Matic L, John D, Lunella FF, Jaé N, Rossbach O, Amrhein C, Sigala F, et al: Adenosine-to-inosine RNA editing controls cathepsin S expression in atherosclerosis by enabling HuR-mediated post-transcriptional regulation. *Nat Med* 2016, 22:1140-1150.

15.	Ramaswami G, Lin W, Piskol R, Tan MH, Davis C, Li JB: Accurate identification of human Alu and non-Alu RNA editing sites. *Nat Methods* 2012, 9:579-581.

16.	Peng Z, Cheng Y, Tan BC-M, Kang L, Tian Z, Zhu Y, Zhang W, Liang Y, Hu X, Tan X, et al: Comprehensive analysis of RNA-Seq data reveals extensive RNA editing in a human transcriptome. *Nat Biotechnol* 2012, 30:253-260.

17.	Li JB, Levanon EY, Yoon J-K, Aach J, Xie B, LeProust E, Zhang K, Gao Y, Church GM: Genome-Wide Identification of Human RNA Editing Sites by Parallel DNA Capturing and Sequencing. *Science (80- )* 2009, 324:1210-1213.

18.	Schaffer AA, Levanon EY: ALU A-to-I RNA Editing: Millions of Sites and Many Open Questions. *Methods Mol Biol* 2021, 2181:149-162.

**Second round of review**

**Reviewer 1**

The authors addressed all my comments.

**Reviewer 2**

The authors address nicely the referees concerns.
